# Metabolomic Analysis Reveals Domestication-Driven Reshaping of Polyphenolic Antioxidants in Soybean Seeds

**DOI:** 10.3390/antiox12040912

**Published:** 2023-04-11

**Authors:** Xuetong Li, Sujuan Li, Jian Wang, Guang Chen, Xiaoyuan Tao, Shengchun Xu

**Affiliations:** 1 Central Laboratory, State Key Laboratory for Managing Biotic and Chemical Threats to the Quality and Safety of Agro-Products, Zhejiang Academy of Agricultural Sciences, Hangzhou 310021, China; lixt@zaas.ac.cn (X.L.); lisj@zaas.ac.cn (S.L.); wangj@zaas.ac.cn (J.W.); chenguang@zaas.ac.cn (G.C.); taoxy@zaas.ac.cn (X.T.); 2 Xianghu Laboratory, Hangzhou 311231, China

**Keywords:** wild soybeans, untargeted metabolomics, domestication-driven reshaping, polyphenolic antioxidants, acylation

## Abstract

Crop domestication has resulted in nutrient losses, so evaluating the reshaping of phytonutrients is crucial for improving nutrition. Soybean is an ideal model due to its abundant phytonutrients and wild relatives. In order to unravel the domestication consequence of phytonutrients, comparative and association analyses of metabolomes and antioxidant activities were performed on seeds of six wild (*Glycine soja* (Sieb. and Zucc.)) and six cultivated soybeans (*Glycine max* (L.) Merr.). Through ultra-high performance liquid chromatography–tandem mass spectrometry (UHPLC-MS/MS), we observed a greater metabolic diversity in wild soybeans, which also displayed higher antioxidant activities. (−)-Epicatechin, a potent antioxidant, displayed a 1750-fold greater abundance in wild soybeans than in cultivated soybeans. Multiple polyphenols in the catechin biosynthesis pathway were significantly higher in wild soybeans, including phlorizin, taxifolin, quercetin 3-*O*-galactoside, cyanidin 3-*O*-glucoside, (+)-catechin, (−)-epiafzelechin, catechin–glucoside, and three proanthocyanidins. They showed significant positive correlations with each other and antioxidant activities, indicating their cooperative contribution to the high antioxidant activities of wild soybeans. Additionally, natural acylation related to functional properties was characterized in a diverse range of polyphenols. Our study reveals the comprehensive reprogramming of polyphenolic antioxidants during domestication, providing valuable insights for metabolism-assisted fortification of crop nutrition.

## 1. Introduction

With the improvement of health and lifestyle standards, crop nutritional quality has received more attention [1]. Crop breeding goals are gradually shifting towards nutrient biofortification [2]. Antioxidant activity is a widely concerned nutritional quality in crops, as it plays a crucial role in preventing various chronic diseases [3]. Antioxidant activity is largely determined by the types and contents of phytochemical antioxidants, such as vitamins, phytosterols, and polyphenols [4,5,6]. Studying phytochemical antioxidants will promote the nutritional improvement of crops to meet people’s health needs. Long-term crop breeding focused on high-yield traits has significantly reduced the diversity of genes related to nutrition quality [7]. Comparative studies of wild and domesticated germplasms will deepen our understanding of domestication-driven reshaping of nutrition traits and accelerate the identification of novel phytonutrient and gene targets for improving cultivated varieties and domesticating potential wild relatives [7].

Soybean, a major staple food worldwide, is not only a valuable source of oil and protein, but also rich in health-promoting small molecules [8]. Isoflavonoids and soyasaponins are well-known phytochemicals in soybeans with a wide range of biological activities in oxidative stress-related dysfunctions [9,10]. Additionally, anthocyanins in pigmented soybeans were generally recognized as a class of star antioxidants [11,12]. Collectively, soybean is a good model crop for investigating the diversity of functional antioxidants and identifying genes related to chemical synthesis and decoration. During the domestication of soybeans, one of the obvious changes is the pigmentation of the seed coats [13]. The anthocyanin biosynthesis-related genes and transcription factors contributed to the pigmented seed coat of soybeans [13,14]. These findings suggest that pigmented wild soybeans exhibit high metabolic diversity in the polyphenolic precursors and products of anthocyanins. Anthocyanins are a well-studied class of flavonoids [15]. Flavonoids are a large class of polyphenols and can be subdivided into flavones, flavonols, flavanones, flavans, flavanol, chalcones, aurones, isoflavones, anthocyanidins, etc. [16]. The multiple phenolic hydroxyl groups on the core backbone of flavonoids contribute to their potent antioxidant activity [17,18]. A series of flavonoids has been applied in health protection and medical treatment fields due to their superior antioxidant feature, such as proanthocyanidins and silymarin [19,20]. In addition to well-studied isoflavones and anthocyanidins, the characterization of domestication consequences of more subclasses of flavonoids will facilitate the exploitation of more polyphenolic antioxidants. Naturally occurring chemical modifications, such as glycosylation and acylation, alter the polarity, solubility, stability, bioavailability, and bioactivity of polyphenolic antioxidants [21,22]. Due to the significant effect of modifications on the functional properties of polyphenolic antioxidants, it is essential to characterize polyphenol modifications to achieve functional improvement.

Given the significance of domestication-driven reshaping of polyphenolic antioxidants in the biofortification of soybean antioxidant capacity, the variation and correlation of metabolomes and antioxidant capacity of wild and cultivated soybeans were comprehensively investigated in our study. The untargeted metabolomics strategy was performed through UHPLC-MS/MS for the global and unbiased analysis of a wide range of soybean phytochemicals. The metabolomes of six wild and six cultivated soybeans were analyzed. The antioxidant activities of identical soybean samples were assessed through the ferric-reducing antioxidant power (FRAP) and the 2,2′-azinobis-(3-ethylbenzothiazoline-6-sulfonate) (ABTS) assays. The multivariate statistical analyses were carried out to screen the featured metabolites and corresponding pathways that were selected during soybean domestication and contributed to the variation of soybean antioxidant capacity. (−)-Epicatechin and diverse types of polyphenols related to catechin biosynthesis displayed significantly higher accumulation levels in wild soybeans. These polyphenols have significant positive correlation relationships with the antioxidant activities of soybean varieties. Furthermore, the characterization of malonylated and acetylated derivatives of various subclasses of polyphenols provides a structural foundation for the functional manipulation of polyphenolic antioxidants. Our study provides a wealth of polyphenolic targets for metabolomics-assisted antioxidant capacity improvement of cultivated soybeans and will boost the development of wild soybeans.

## 2. Materials and Methods

### 2.1. Reagents and Plant Materials

Chromatographic-grade methanol and acetonitrile were purchased from Merck (Darmstadt, Germany). Chromatographic-grade acetic acid was purchased from Sigma-Aldrich (Darmstadt, Germany). Distilled water was purchased from Watsons (Guangzhou, China). Chemical standards were purchased from ANPEL Laboratory Technologies Inc. (Shanghai, China), Shanghai yuanye Bio-Technology Company, Ltd. (Shanghai, China), and Macklin Inc. (Shanghai, China).

Six cultivated soybean varieties (*Glycine max*), including Qi huang No. 34, Yu dou No. 22, Wan dou No. 28, Ji dou No. 17, Jin dou No. 23, and Tong shan tian e dan, and six wild soybean varieties (*Glycine soja*), including W1, W11, W55, W61, W71, and W92, were used in this study. Soybean varieties were planted and harvested from July to October 2020 at the field of Yangdu base of Haining, Zhejiang, China (E 120°423′, N 30°444′). The average altitude in relation to sea level of the planting field is 5 m. The average temperature and humidity during the growth of soybean varieties were 24 °C and 82%, respectively. An herbicide named acetochlor (CAS: 34256-82-1) was used from sowing to seedling emergence. An insecticide named imidacloprid (CAS: 138261-41-3) was used at the seedling stage. There were three independent repetitions for each soybean accession. Two grams of dried mature grains were thoroughly ground with a tissue grinder at 55 Hz for 40 s. The sample powder was stored at −80 °C for further processing.

### 2.2. Metabolite Extraction

Briefly, the metabolites of 150 mg of soybean grains powders were extracted by 1.5 mL 70% aqueous methanol solution. The solution was vortexed three times for 10 min each session and placed in a 4 °C refrigerator overnight. The solution was centrifuged at 12,000× *g* for 10 min at 4 °C. The supernatant of the solution was dried using a concentrator under vacuum and redissolved with 150 μL 70% aqueous methanol solution containing 20 ng/μL dihydrocapsaicin as internal standard. The solution was filtered using a 0.22 μm needle filter and transferred into a chromatographic sample bottle for UHPLC-MS/MS analysis.

### 2.3. UHPLC-MS/MS Analysis

Liquid chromatographic analysis of samples was performed using a Thermo Scientific Dionex Ultimate 3000 RSLC (HPG) ultra-performance liquid chromatography (ThermoFisher Scientific, Waltham, MA, USA) with a Waters ACQUITY UPLC BEH C18 column (1.7 μm, 2.1 mm × 100 mm, Waters Corporation, Milford, MA, USA). The mobile phase consisted of (A) water with 0.04% acetic acid and (B) acetonitrile with 0.04% acetic acid. The gradient program was set to the following parameters: 95:5 A/B at 0 min, 5:95 A/B at 20.0 min, 5:95 A/B at 24.0 min, 95:5 A/B at 24.1 min, and 95:5 A/B at 30 min. The flow rate was 0.3 mL/min. The injection volume was 3 μL.

Mass spectrometry analysis was performed using a Thermo Fisher Q Exactive hybrid Q-Orbitrap-high-resolution mass spectrometer (ThermoFisher Scientific, Waltham, MA, USA) coupled with a UPLC system. The full MS/dd-MS2 mode was applied in MS acquisition. MS acquisition was performed in positive and negative ion modes. The *m/z* range of the full MS scan was 100 to 1200 *m/z*. The mass resolution of the full MS scan was 70,000 at *m/z* 200. The mass resolution of data-dependent MS/MS was 17,500 at *m/z* 200.

The process for heated electrospray ionization (HESI) was set to the following parameters: spray voltage (+), 3500 V; spray voltage (−), 2800 V; capillary temperature, 275 °C; sheath gas, 40 arb; aux gas, 10 arb; probe heater temperature, 350 °C; s-lens RF level, 50. The energies of higher energy collisional dissociation (HCD) were 20, 40, and 60 eV, and the average MS/MS spectrum was obtained.

The mixture of grains obtained from all soybean varieties was used as the reference mixture for quality control. The reference mixture was submitted to UHPLC-MS/MS system before and after every 18 samples.

### 2.4. Mass Spectrometric Data Processing

Raw mass spectrometric data were analyzed using Compound Discoverer™ software (Version 3.3, ThermoFisher Scientific, Waltham, MA, USA) via its automatic workflow. The process for aligning retention times was set to the following parameters: mass tolerance, 5 ppm; maximum shift, 0.5 min. The process for detecting compounds was set to the following parameters: min peak intensity, 1E6; signal-to-noise ratio (S/N) threshold, 5. The in-house script based on the Xcalibur development kit of Xcalibur™ software (version 2.2, ThermoFisher Scientific, Waltham, MA, USA) was developed to automatically extract MS/MS spectra of detected metabolic features.

### 2.5. Structural Annotation and Identification of Metabolites

The structural annotation of metabolites was implemented in our previous study [23]. The first annotation approach utilized the experimental reference mass spectra library for retrieval and similarity matching. The experimental reference mass spectra were collected and integrated from MassBank, ReSpect, RIKEN PlaSMA, Fiehn HILIC, and Vaniya-Fiehn Natural Products libraries. The INCOS algorithm was implemented for similarity scoring between query and reference mass spectra. The second approach was developed to extend annotation coverage via a theoretical reference mass spectra library. A structural database of biologically relevant compounds (SDBRC) constructed in our previous study was used as a reference to retrieve and generate theoretical mass spectra. CFM-ID software (Version 4) [24] was utilized for theoretical mass spectra prediction of compounds from SDBRC and similarity scoring between query and theoretical reference mass spectra.

The annotation results of featured metabolite features were further identified by comparing the *m/z* of precursor, retention time, and fragmentation pattern with standard compounds.

Unknown MS/MS spectra were further structurally characterized using characteristic fragment ions of aglycone and featured neutral loss of modification groups based on published literature.

### 2.6. Assessment of Total Antioxidant Capacity

#### 2.6.1. The FRAP Assay

The total antioxidant capacities of 6 cultivated and 6 wild soybean varieties were assessed through the FRAP assay with the reagent kit (Cominbio biological technology Co., Ltd., Suzhou, China). The ability of antioxidants in reducing a ferric–tripyridyltriazine (Fe^III^-TPTZ) complex to the ferrous (Fe^II^) form under acidic conditions reflects the total antioxidant capacity [25]. The blue color of Fe^II^-TPTZ with an absorption maximum at 593 nm detection wavelength was used to quantitate the total antioxidant capacity. A total of 100 mg of soybean grain powders were fully mixed and extracted with 1 mL extracting solution. The mixed solution was centrifuged at 10,000× *g* for 10 min at 4 °C. The supernatant of the mixed solution was used for the next testing. A total of 10 uL of the testing sample was mixed and reacted with 190 uL mixed reagent of the reagent kit in 96-well plates for 20 min. Trolox was used as a reference to measure the total antioxidant capacities of samples. The total antioxidant capacities of samples were acquired by detecting the absorbance through a microplate reader at 593 nm.

#### 2.6.2. The ABTS Assay

The total antioxidant capacities of 6 cultivated and 6 wild soybean varieties were assessed through the ABTS assay with the reagent kit (Cominbio biological technology Co., Ltd., Suzhou, China). The ability of antioxidants in scavenging the ABTS free radicals reflects the total antioxidant capacity [26]. The color change after the reaction with an absorption maximum at 734 nm detection wavelength was used to quantitate the total antioxidant capacity. A total of 100 mg of soybean grains powders were fully mixed and extracted with 1ml extracting solution. The mixed solution was centrifuged at 10,000× *g* for 10 min at 4 °C. The supernatant of the mixed solution was used for the next testing. A total of 10 uL of the testing sample was mixed and reacted with 190 uL mixed reagent of the reagent kit in 96-well plates. Trolox was used as a reference to measure the total antioxidant capacities of the samples. The total antioxidant capacities of the samples were acquired by detecting the absorbance through a microplate reader at 734 nm within 10 min.

### 2.7. Statistical Analysis

Principal component analysis (PCA) and orthogonal partial least squares discriminant analysis (OPLS-DA) were carried out by SIMCA-P software (version 14.0, Umetrics, Umea, Sweden). The variable importance in projection (VIP) value of the OPLS-DA model was used as a decisive indicator, and a VIP value greater than 1.5 was defined as a threshold for the selection of featured metabolite with the significant inter-group difference. The heatmap was plotted by pheatmap package in the R environment. The correlation analysis was implemented in the R environment. A *p*-value less than 0.01 was defined as a threshold for the selection of significant correlation.

## 3. Results

### 3.1. Comparative Analysis Revealed the Metabolome Variances between Wild and Cultivated Soybeans

**Figure 1:**
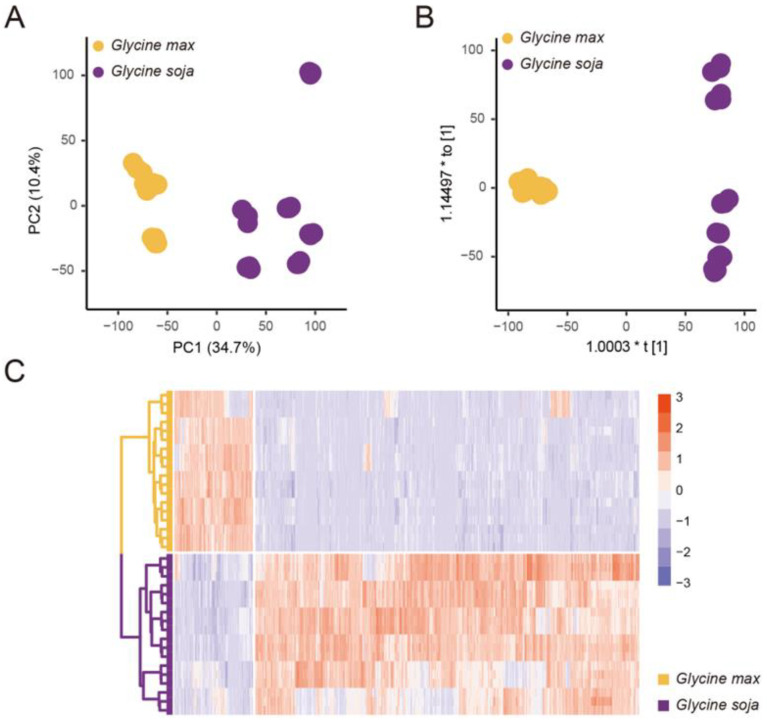
The metabolic variation between wild and cultivated soybeans. Shapes in purple indicate wild soybean varieties (*Glycine soja*). Shapes in yellow indicate cultivated soybean varieties (*Glycine max*). (**A**) The score plot for principal component analysis of metabolic profiles of wild and cultivated soybean varieties. PC1, the first principal component. PC2, the second principal component. (**B**) The score plot for OPLS-DA of metabolic profiles of wild and cultivated soybean varieties. (**C**) The heatmap of relative abundance of featured metabolites of wild and cultivated soybean varieties. The values of relative abundance were centered and scaled.

To define the metabolic variance of soybean during domestication, the untargeted metabolomics analysis of mature seeds of six wild and six cultivated soybean varieties was performed using UHPLC-MS/MS. A total of 7802 and 7175 metabolite features were acquired using positive and negative ion modes, respectively. Metabolite features were structurally annotated with reference libraries containing high-quality experimental and theoretical mass spectra. The annotation results of important metabolite features were further identified by comparing them with chemical standards. A PCA approach was applied to assess the metabolite abundance variation (Figure 1A). The wild and cultivated soybeans were classified into two distinct clusters. The first principal component (PC1) and the second principal component (PC2) account for 34.7% and 10.4% of the metabolic variance, respectively. The wild soybeans showed higher variance among diverse varieties compared with cultivated soybeans, indicating the high level of metabolic diversity of wild soybeans. The OPLS-DA approach was further used to explore the featured metabolites that contributed to the metabolic variations (Figure 1B). The VIP value generated from the OPLS-DA model is a decisive indicator, and VIP values greater than 1.5 were defined as the featured metabolites. A total of 83% of the featured metabolites displayed significantly higher abundance in wild soybeans compared with cultivated soybeans (Figure 1C). The average and median of the fold-changes of featured metabolites in wild soybeans than in cultivated soybeans are 26.11 and 10.92, respectively. These results indicated that the abundance of massive metabolites was acutely reduced during soybean domestication.

### 3.2. Dissection of the Key Featured Metabolites of Soybean Domestication

**Figure 2:**
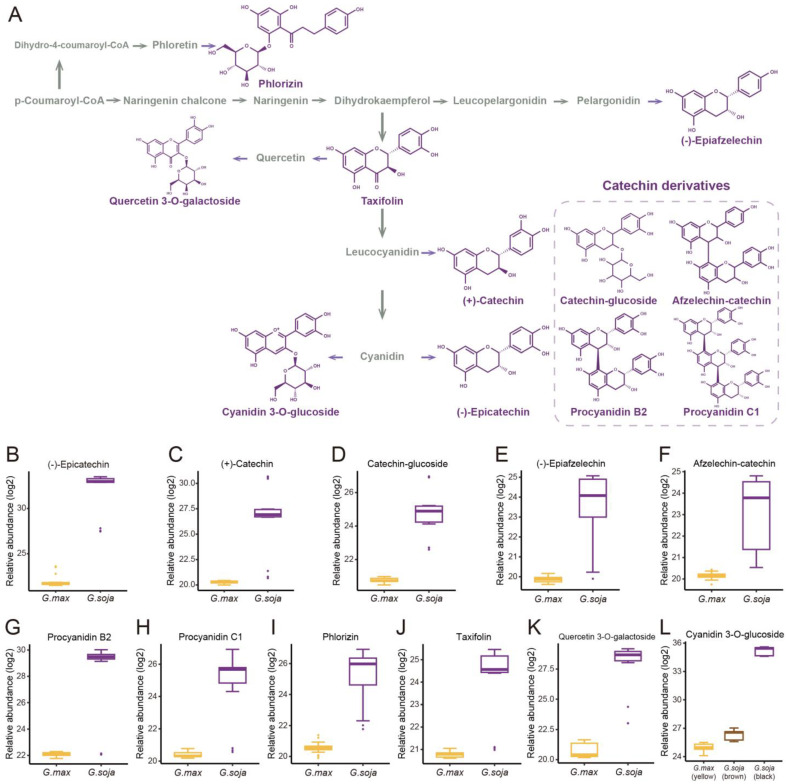
The domestication consequence of polyphenols in catechin biosynthesis pathway. Boxes in purple indicate wild soybean varieties (*Glycine soja*, *G. soja*). Boxes in yellow indicate cultivated soybean varieties (*Glycine max*, *G. max*). The relative abundance was log2-transformed. (**A**) The structure and position of featured polyphenols in the catechin biosynthesis pathway. This diagram was plotted according to KEGG PATHWAY Database (ID: map00941). (**B**) The relative abundance of (−)-epicatechin in wild and cultivated soybean varieties. (**C**) The relative abundance of (+)-catechin in wild and cultivated soybean varieties. (**D**) The relative abundance of catechin–glucoside in wild and cultivated soybean varieties. (**E**) The relative abundance of (−)-epiafzelechin in wild and cultivated soybean varieties. (**F**) The relative abundance of afzelechin–catechin in wild and cultivated soybean varieties. (**G**) The relative abundance of procyanidin B2 in wild and cultivated soybean varieties. (**H**) The relative abundance of procyanidin C1 in wild and cultivated soybean varieties. (**I**) The relative abundance of phlorizin in wild and cultivated soybean varieties. (**J**) The relative abundance of taxifolin in wild and cultivated soybean varieties. (**K**) The relative abundance of quercetin 3-*O*-galactoside in wild and cultivated soybean varieties. (**L**) The relative abundance of cyanidin 3-*O*-glucoside in wild and cultivated soybean varieties.

The phytochemical and pathway distribution of featured metabolites were fully investigated for a better understanding of the domestication consequence on the soybean metabolome. A series of polyphenols in the pathway of catechin biosynthesis were found to prominently contribute to the metabolome variance during domestication (Figure 2A, Table 1 and Table S1).

(−)-Epicatechin, a tetrahydroxy flavan-3-ol, was found to be significantly higher in wild soybean varieties (Figure 2B) and accounted for the largest metabolic variance with a fold-change of 1750 and a VIP value of 3.41, implying that the extreme reshaping has occurred on the catechin synthetic pathway during domestication. (+)-Catechin also displayed significantly higher abundance in wild soybeans (Figure 2C). In addition, the glycosylated derivative of catechin (catechin–glucoside) was found to be significantly higher in wild soybeans (Figure 2D). (−)-Epiafzelechin belongs to trihydroxy flavan-3-ols and is a prominent catechin derivative. (−)-Epiafzelechin was found to display significantly higher abundance in wild soybean varieties in our study (Figure 2E). The three catechin oligomers belonging to proanthocyanidins, afzelechin–catechin, procyanidin B2 (catechin dimer), and procyanidin C1 (catechin trimer), were found to be significantly higher in wild soybean varieties in our study (Figure 2F–H).

Besides the derivatives of catechins, a variety of polyphenols in the upstream pathway of catechin biosynthesis were significantly selected during soybean domestication. The significantly higher accumulation levels of phlorizin were observed in wild soybeans (Figure 2I). Phlorizin is a prominent member of glycosylated dihydrochalcones and is located at the upstream pathway of catechin biosynthesis. Phlorizin is specifically enriched in the Malus species and shows low amounts in a few other species [28]. Notably, the distribution of phlorizin in wild soybeans was confirmed in our study. Taxifolin (dihydroquercetin) is a natural dihyroflavonol and was found to be significantly enriched in wild soybeans in our metabolic analysis (Figure 2J). In the previous study, taxifolin has been dissected as a core metabolic precursor in catechin biosynthesis due to its significant effect on metabolic flux regulation [29]. The concordant high accumulation levels of taxifolin, (+)-catechin, and (−)-epicatechin in wild soybeans indicate their close interactions in the biosynthesis pathway. Quercetin 3-*O*-galactoside is a glycosylated derivative of quercetin, a direct product of taxifolin. The significant high accumulation level of quercetin 3-*O*-galactoside was observed in wild soybeans in our study (Figure 2K). Cyanidin is a major component of anthocyanidins and a direct substrate of (−)-epicatechin. The glycosylated form of cyanidin (cyanidin 3-*O*-glucoside) was found to specifically accumulate in black wild soybean varieties while showing lower abundance in brown wild and yellow cultivated varieties in our study (Figure 2L). All results suggest the occurrence of domestication-driven remodeling of the polyphenolic biosynthesis pathway.

### 3.3. Correlation Analysis between Domestication-Related Polyphenols and Antioxidant Activities of Soybeans

**Figure 3:**
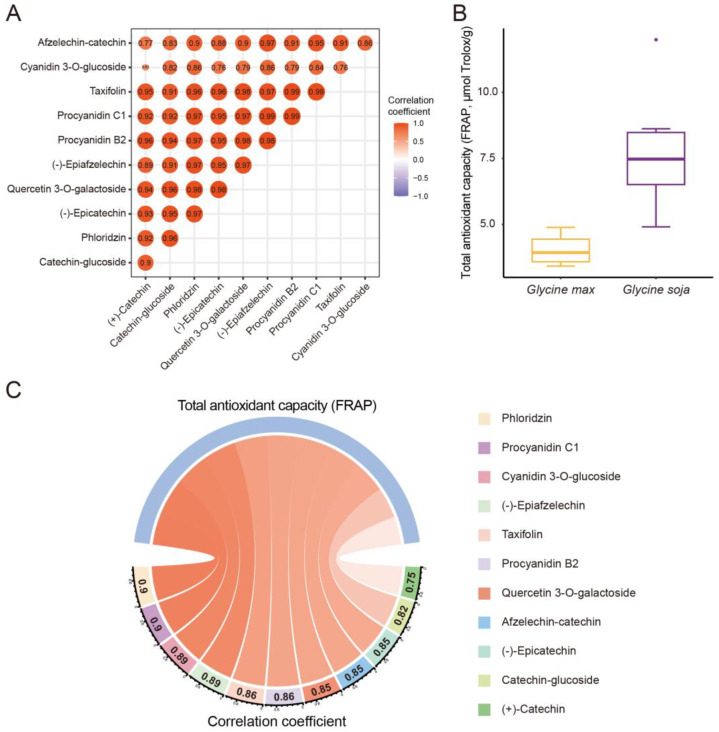
The significant correlations between domestication-related polyphenols and total antioxidant capacities of soybeans. (**A**) The significant positive correlations between domestication-related polyphenols. (**B**) The significant variation of total antioxidant capacity determined by the FRAP assay between wild (*Glycine soja*) and cultivated soybean varieties (*Glycine max*). Student’s *t*-test was used. (**C**) The chord diagram of strong positive correlative relationship between each domestication-related polyphenols and total antioxidant capacities determined by the FRAP assay.

In order to investigate the relationships between domestication-related polyphenols, a correlation analysis was performed on the abundance of domestication-related polyphenols among six wild soybeans and six cultivated soybean varieties. All of the domestication-related polyphenols showed significant positive correlations *(p*-value < 0.01) with each other. The correlation coefficients range from 0.63 to 0.99 with a median of 0.95 (Figure 3A). The strong correlations between diverse domestication-related polyphenols imply their close interactions in biosynthesis and provide further evidence for the comprehensive reshaping of the polyphenolic biosynthesis pathway.

In order to build a bridge of metabolite and nutrient traits of soybeans, the total antioxidant capacity of six cultivated and six wild soybean varieties was assessed through the FRAP and ABTS assays. The significantly (*p*-value < 0.05) higher value of total antioxidant capacity was observed through the FRAP assay in wild soybean varieties compared with cultivated soybean varieties (Figure 3B, Table S2). Similarly, the significantly (*p*-value < 0.01) higher value of total antioxidant capacity was observed through the ABTS assay in wild soybean varieties compared with cultivated soybean varieties (Figure S3A, Table S2). The higher total antioxidant capacities of wild soybeans were consistent with their higher metabolic diversity. Furthermore, the associations between the polyphenolic abundances and the total antioxidant capacities of six wild soybeans and six cultivated soybean varieties have been investigated through correlation analysis. Significant positive correlations (*p*-value < 0.01) have been observed between the total antioxidant capacities from the FRAP assay and the abundance of all of the domestication-related polyphenols with correlation coefficients ranging from 0.75 to 0.9 (Figure 3C). In the ABTS assay, similar significant positive correlations (*p*-value < 0.01) have been observed with correlation coefficients ranging from 0.74 to 0.9 (Figure S3B). The strong correlations suggest the significant contributions of domestication-related polyphenols to the variation of the total antioxidant capacities between wild and cultivated soybeans. These domestication-related polyphenols can be subdivided into dihydrochalcones, dihydroflavonols, flavonols, anthocyanins, flavanols, and proanthocyanidins. Our results indicate that the diverse types of polyphenols jointly contributed to the high antioxidant activities of wild soybeans. Phlorizin, belonging to dihydrochalcones, was known for its superior antioxidant, regulation of glucose transport, and anti-inflammatory properties [30]. Taxifolin is a natural dihydroflavonol and has been characterized as a potent antioxidant. Taxifolin displays multiple pharmacological activities in inflammation, cardiovascular, and other oxidative stress-related diseases [31,32]. Quercetin 3-*O*-galactoside is a glycosylated flavonol. The functional activities of quercetin 3-*O*-galactoside in antioxidation, anti-inflammation, and antidepressants have been confirmed by a series of research [33,34,35]. Cyanidin 3-*O*-glucoside belongs to anthocyanins and has been proven to display powerful antioxidant activity. The two phenolic hydroxyl groups on the B ring of its structure potentially contribute to its antioxidant activity [36]. (−)-Epicatechin and (+)-catechin, belonging to flavanols, show excellent antioxidant properties and a variety of preventing and protecting functions in oxidative stress-related dysfunctions, such as cardiovascular disease, diabetes, and neurodegenerative disease [17,37]. The glycosylated form of catechin (catechin–glucoside) can improve the stability and absorbability of catechin [22]. As the structural variant of (−)-epicatechin, (−)-epiafzelechin similarly displays potent antioxidant activity [38]. The protection effects of (−)-epiafzelechin in estrogen deficiency-induced bone loss and inflammation have been confirmed in previous studies [39,40]. Afzelechin–catechin, procyanidin B2, and procyanidin C1 belong to proanthocyanidins. Procyanidin B2 has been characterized as a potent antioxidant and has an inhibition effect on vascular calcification [20]. Procyanidin C1 has potent antioxidant activity and has been validated as a natural senotherapeutic agent in age-related chronic pathologies due to its specific effects on senescent cells [41].

### 3.4. Panoramic Malonylation Modificome of Flavonoids and Isoflavonoids

**Figure 4:**
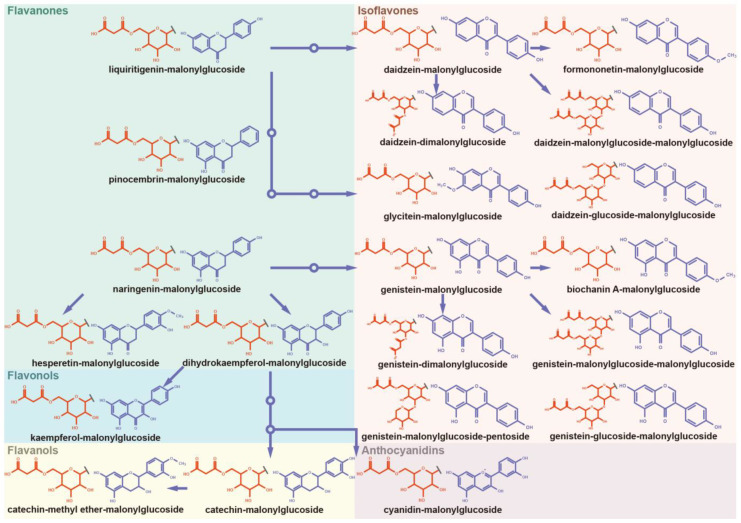
Panorama of malonylglycosylation of flavonoids and isoflavonoids. The malonylglycosylated derivatives and biosynthesis pathway of diverse flavanones, flavonols, flavanols, anthocyanidins, and isoflavones aglycones was plotted according to KEGG PATHWAY Database (IDs: map00941 and map00943).

Modification of polyphenolic antioxidants, such as glycosylation and acylation, can alter their metabolic characteristics, bioavailability, and bioactivity [21,22,42]. It is essential to systematically investigate the naturally occurring modifications of polyphenols for the functional manipulation of antioxidants. In soybeans, malonylation is a widespread modification in isoflavonoids, which plays a key role in the nodulation and response to oxidative-related abiotic stresses [43,44]. In previous studies of soybean, the characterization of malonylation mainly focused on isoflavonoids. The malonylation modificome of the diverse subclasses of polyphenols, such as flavones, flavonols, flavans, and flavanols, remains poorly studied. To provide new insight into the diversity of naturally occurring malonylation of polyphenols, the characteristic fragment ion and neutral loss of MS/MS spectra in soybean metabolome were fully analyzed. A series of malonyl-conjugates of flavonoids and isoflavonoids distributed over a wide range of biosynthetic pathways were found in our study (Figure 4, Table S3 [27,45,46,47,48,49,50,51,52]).

Liquiritigenin (dihydroxyflavanone) and naringenin (trihydroxyflavanone) are key linkages of flavonoid and downstream isoflavonoid biosynthesis pathways. Liquiritigenin–malonylglucoside and naringenin–malonylglucoside were newly uncovered in our study (Figure S4A,B). In addition, the malonylglycosylated derivative of pinocembrin (the regioisomer of liquiritigenin) was newly uncovered (Figure S4C). Hesperetin (trihydroxy-methoxyflavanone) and dihydrokaempferol (trihydroxy-dihyroflavonol) are the direct derivatives of naringenin in the polyphenolic biosynthetic pathway. The malonylglycosylated derivatives of hesperetin and dihydrokaempferol were newly characterized (Figure S4D,E). Kaempferol (trihydroxyflavonol) was derived from dihydrokaempferol through flavonol synthase and its malonylglycosylated form (kaempferol–malonylglucoside) was characterized (Figure S4F). Cyanidin belongs to anthocyanidins, and its malonylglucoside-conjugate was observed in soybean seeds (Figure S4G). Catechin and catechin-methyl ether belong to flavanols, and their malonylglycosylated derivatives were newly uncovered in soybean seeds (Figure S4H,I). The ubiquitous malonylation of diverse types of polyphenols indicates the occurrence of malonyltransferases with high reactivity and diverse substrate specificity in soybeans. The structural insights gained from our findings will facilitate the exploration of malonyltransferases, which play a crucial role in modifying the structure and function of polyphenolic antioxidants.

In the isoflavonoid biosynthetic pathway, the general malonylglycosylated derivatives of a series of isoflavones have been characterized, such as daidzein–malonylglucoside, formononetin–malonylglucoside, genistein–malonylglucoside, biochanin A–malonylglucoside, and glycitein–malonylglucoside (Table S3). In addition, the diverse combination patterns of the malonylglycosylated derivatives of daidzein and genistein were uncovered in our study, such as daidzein–dimalonylglucoside, genistein–dimalonylglucoside, daidzein–glucoside–malonylglucoside, genistein–glucoside–malonylglucoside, genistein–malonylglucoside–pentoside, daidzein–malonylglucoside–malonylglucoside, and genistein–malonylglucoside–malonylglucoside (Table S3).

### 3.5. Panoramic Acetylation Modificome of Flavonoids and Isoflavonoids

**Figure 5:**
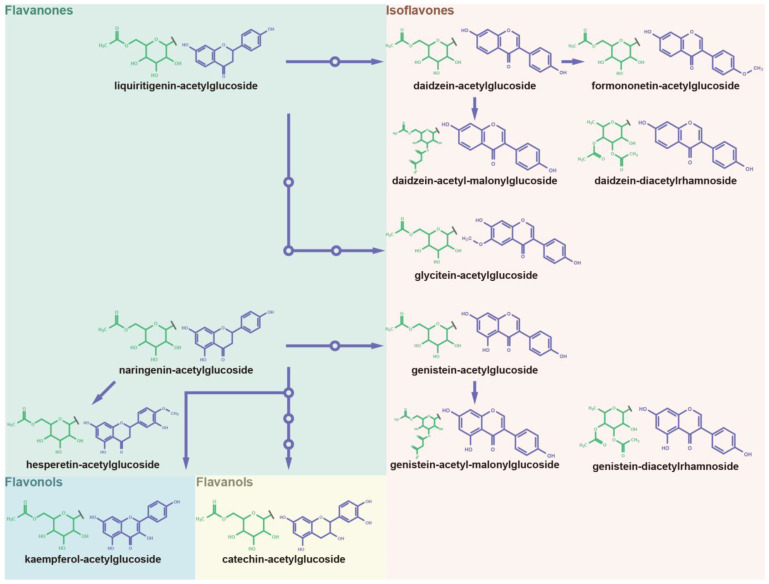
Panorama of acetylglycosylation of flavonoids and isoflavonoids. The acetylglycosylated derivatives and biosynthesis pathway of diverse flavanones, flavonols, flavanols, and isoflavones aglycones was plotted according to KEGG PATHWAY Database (IDs: map00941 and map00943).

Acetyl isoflavones are a group of ubiquitous polyphenols in soybeans [53]. Besides acetyl isoflavones, the acetylated derivatives of a series of polyphenols widely distributed throughout the biosynthetic pathway were investigated in our study (Figure 5, Table S3). The acetylglycosylated conjugates of naringenin and liquiritigenin belonging to flavanones were characterized in our study (Figure S5A,B). The acetylglycosylated derivatives of hesperetin and kaempferol were further characterized (Figure S5C,D). The catechin–acetylglucoside was newly uncovered in soybean seeds in our study (Figure S5E). In the isoflavonoid biosynthetic pathway, the common acetylglycosylated conjugates of daidzein, formononetin, genistein, and glycitein have been characterized (Table S3). Moreover, the novel acetylated patterns of daidzein and genistein were uncovered in our study, such as daidzein–diacetylrhamnoside, genistein–diacetylrhamnoside, daidzein–acetyl-malonylglucoside, and genistein-acetyl-malonylglucoside (Table S3). The aglycones of acetylation overlap with malonylation in soybeans, indicating the widespread and concurrent acylation of polyphenols.

## 4. Discussion

With the improvement of people’s living and health standards, the nutritional quality of crops has attracted more research attention [1]. The domestication process of crops generally resulted in a loss of nutritional quality [7]. The undomesticated wild crop has been characterized as a precious resource for crop nutrition improvement [7]. Wild soybean is the progenitor of modern cultivated soybean and displays high genetic diversity [14]. The comprehensive study of metabolic consequences of soybean domestication will facilitate the characterization of phytonutrient and pathway targets for nutrition improvement. However, the investigation of soybean domestication effects mainly focuses on the morphological traits and energy substances [54,55,56]. There still needs to be more efforts to explore the domestication-driven reshaping of phytonutrients.

In our study, untargeted metabolomic analysis was performed to define the domestication consequence of the soybean metabolome. The antioxidant activity was assayed to typically characterize the variation of nutritional quality traits between wild and cultivated soybeans. Diverse types of polyphenols related to catechin biosynthesis were found to be prominently selected during domestication. The significant contributions of these polyphenols to the superior antioxidant activity of wild soybean were characterized through correlation analysis. Moreover, panoramic malonylation and acetylation modificomes were characterized in soybean, providing the structural basis for the functional improvement of polyphenolic antioxidants.

### 4.1. Domestication-Related Polyphenols, the Promising Targets for Nutrition Fortification

As the downstream product of the flavonoid biosynthetic pathway, (−)-epicatechin accounted for the largest abundance variation between wild and cultivated soybeans. The domestication consequence of polyphenols in the upstream catechin biosynthetic pathway (phlorizin, taxifolin, quercetin 3-*O*-galactoside, and cyanidin 3-*O*-glucoside) were comprehensively defined. Furthermore, the significant variation in structural variants of (−)-epicatechin ((+)-catechin and (−)-epiafzelechin) and catechin derivatives (catechin–glucoside, afzelechin–catechin, procyanidin B2, and procyanidin C1) was uncovered in our study. Catechins are usually present in the seed coats of pigmented legumes, such as lentils and cranberry beans [57,58,59]. In the previous study, three major polyphenols, including epicatechin, cyanidin 3-*O*-glucoside, and delphinidin 3-*O*-glucoside were isolated and identified from the seed of wild soybeans [60]. Epicatechin was suggested to be functionally related to seed hardness and germination traits in wild soybeans [60]. In further research, the distinctive accumulation of epicatechin has been observed in the seeds of *Glycine soja* Dolkong and *Glycine max* Napjakong (semi-wild cultivar) rather than Glycine max Hwangkeum (domesticated cultivar) [61]. Likewise, higher accumulation levels of three major antioxidants (cyanidin-3-*O*-glucoside, procyanidin B2, and epicatechin) were observed in wild black soybeans compared with cultivated black soybeans [62]. Additionally, the metabolic variation has been investigated in the leaves of cultivated (*Glycine max*) and semi-wild (*Glycine gracilis*) soybean at different growth stages. Rutin and its precursor (quercetin-3-*O*-glucoside) were detected only in *Glycine gracilis* rather than *Glycine max* [63]. These previous studies highlight the natural variation of some polyphenols in diverse species of soybean. Importantly, the comprehensive domestication-driven reshaping of a wide subclass of polyphenols was further dissected by our study, including dihydrochalcones, dihydroflavonols, flavonols, anthocyanins, flavanols, and proanthocyanidins. Our study uncovered a closely related connection between these polyphenols, providing further evidence of their interactions at the pathway level. The dissection of panoramic domestication-driven variations of diverse types of polyphenols associated with catechin biosynthesis will facilitate catechin biofortification in dietary soy products. Meanwhile, the high accumulation level of phlorizin in wild soybeans was newly observed in our study, which will boost the exploitation and fortification of phlorizin from soybean sources as dietary supplements. The significant accumulations of taxifolin and its derivative (quercetin 3-*O*-galactoside) in wild soybeans provide further evidence for the high nutritional quality of wild soybeans. The results of correlation analysis indicate the synergetic contribution of these polyphenols to the high antioxidant activities of wild soybeans. A wide variety of antioxidation-related polyphenols belonging to dihydrochalcones, dihydroflavonols, flavonols, anthocyanins, flavanols, and proanthocyanidins can be used as the combined metabolic targets for directed improvement of soybean antioxidant activity and precise screening of excellent germplasm resources.

### 4.2. Panoramic Insights into Malonylome and Acetylome of Polyphenols

The comprehensive investigation of malonylation is important for the functional improvement of polyphenolic antioxidants. However, beyond isoflavones, the malonylation modificome of other subclasses of polyphenols has not been well studied. Advances in UHPLC-MS/MS facilitate the high-throughput mining of plant chemical modificome on a large scale [64]. In our study, benefited from UHPLC-Q-Exactive orbitrap mass spectrometry and corresponding computational approach, malonylated flavonoids of diverse types were characterized, including five flavanones, one flavonol, two flavanols, one anthocyanidin, and twelve isoflavones. In our study, the malonylation diversity was expressed as three aspects: (1) subclass diversity. The aglycone types of malonylated derivatives covered a large proportion of subclasses of flavonoids and isoflavonoids; (2) pathway relationship diversity. The aglycones of the malonylated derivatives were spread throughout the upstream and downstream pathways of flavonoid and isoflavonoid biosynthesis; and (3) combination pattern diversity. Dimalonylglycosylation, glycosylation combined with malonylglycosylation, and double malonylglycosylation were observed in isoflavone aglycone (daidzein and genistein). The malonylation diversity of flavonoids and isoflavonoids indicates the diversity and broad substrate specificity of malonyltransferase in soybeans.

In addition, our study revealed a series of acetylated flavonoids and isoflavonoids, including three flavanones, one flavonol, one flavanol, and eight isoflavones. The flavonoid and isoflavonoid aglycones of acetylation overlapped with the malonylation. This finding indicates that acetyltransferases and malonyltransferases are consistently active in soybeans. In the current studies, the biosynthetic pathways of aglycone of flavonoids and isoflavonoids were extensively studied, while enzymes and regulatory factors related to modification reactions need to be further explored. Our study revealed a wide range of malonylated and acetylated polyphenols, which provide valuable insights into the functional exploration of modification enzymes for the improvements of the bioactivity and bioavailability of polyphenolic antioxidants in soybean products.

## 5. Conclusions

In conclusion, a metabolic reprogramming of the catechin biosynthetic pathway was uncovered in soybean, indicating a significant selection of antioxidation-related polyphenols during domestication. Moreover, the malonylation and acetylation diversity of polyphenols at the subclass, pathway relationship, and combination pattern levels were characterized, which implies the broad substrate specificity and high catalytic activity of malonyltransferase and acetyltransferase with physiological and pharmacological significance to polyphenolic antioxidants in soybean. The domestication selection polyphenolic targets and panoramic malonylation and acetylation modificomes revealed by our study will boost the metabolism-assisted improvement of cultivated soybean and utilization of wild soybean to improve the nutritional and economical value of soybean products.

## Figures and Tables

**Table 1:** Detailed information on domestication-related polyphenols.

Name	Structure	Classification	RT (min)	Exact Mass	Characteristic Fragment Ions	Reference
cyanidin 3-*O*-glucoside	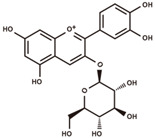	anthocyanins	5.25	449.1084	[M–Glc]^+^: 287.0556, ^0,2^A^+^: 149.0239, ^0,2^B^+^: 137.0239, ^0,3^A^+^: 121.029.	Chemical standard
(+)-catechin	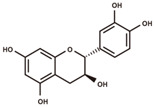	flavanols	5.51	290.079	[M+H]^+^: 291.0869, ^1,4^B^+^: 165.0552, ^1,4^B^+^–H_2_O: 147.0446, ^1,3^A^+^: 139.0395, ^1,2^B^+^: 123.0446.	Chemical standard
catechin-glucoside	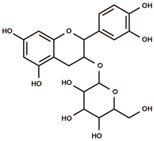	flavanols	5.69	452.1319	[M+H–Glc]^+^: 291.0869, ^1,4^B^+^: 165.0552, ^1,2^A^+^–H_2_O: 151.0395, ^1,4^B^+^–H_2_O: 147.0446, ^1,3^A^+^: 139.0395, ^1,2^B^+^: 123.0446.	Fragmentation pattern in [27].
procyanidin B2	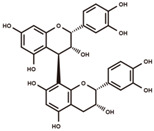	proanthocyanidins	6.03	578.1424	[M+H]^+^: 579.1503, 427.1029, 409.0923, 301.0712, 291.0869, 289.0712, 287.0556, 165.0552, 151.0395, 147.0446, 139.0395, 127.0395, 123.0446.	Chemical standard
(−)-epicatechin	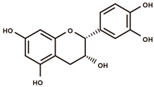	flavanols	6.32	290.079	[M+H]^+^: 291.0869, ^1,4^B^+^: 165.0552, ^1,4^B^+^–H_2_O: 147.0446, ^1,3^A^+^: 139.0395, ^1,2^B^+^: 123.0446.	Chemical standard
procyanidin C1	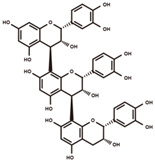	proanthocyanidins	6.55	866.2058	[M+H]^+^: 867.2136, 589.1346, 579.1503, 451.1029, 427.1029, 409.0923, 301.0712, 291.0869, 289.0712, 287.0556, 165.0552, 151.0395, 147.0446, 139.0395, 127.0395, 123.0446.	Chemical standard
afzelechin-catechin	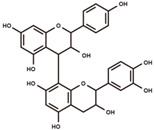	proanthocyanidins	6.61	562.1475	[M+H]^+^: 563.1553, 411.108, 291.0869, 287.0556, 285.0763, 273.0763, 165.0552, 151.0395, 147.0446, 139.0395, 123.0446, 107.0497.	Fragmentation pattern in [27].
(−)-epiafzelechin	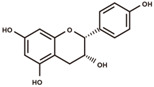	flavanols	7.00	274.0841	[M+H]^+^: 275.0919, ^1,4^B^+^: 149.0603, ^1,3^A^+^: 139.0395, ^1,2^B^+^: 107.0497.	Chemical standard
quercetin 3-*O*-galactoside	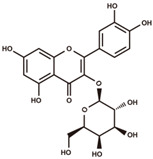	flavonols	7.39	464.0955	[M+H–Gal]^+^: 303.0505, [M+H–Gal–H_2_O]^+^: 285.0399, ^0,2^A^+^: 165.0188, ^1,3^A^+^: 153.0188, ^0,2^B^+^: 137.0239.	Chemical standard
taxifolin	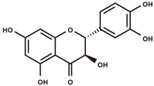	dihydroflavonols	7.74	304.0583	[M–H]^−^: 303.0505, [M–H–H_2_O]^−^: 285.0399, ^1,4^B^−^: 177.0188, ^1,3^A^−^: 151.0031, ^1,4^A^−^: 125.0239.	Chemical standard
phlorizin	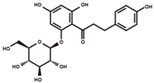	dihydrochalcones	8.55	436.1369	[M–H–Glc]^−^: 273.0763, [M–H–Glc–C_7_H_6_O]^−^: 167.0344, [M–H–Glc–C_7_H_6_O–C_2_H_2_O]^−^: 125.0239, [M–H–Glc–C_7_H_6_O–CO_2_]^−^: 123.0446.	Chemical standard

[M+H]^+^: the protonated precursor ion of polyphenols. [M–H]^−^: the deprotonated precursor ion of polyphenols. ^i,j^A^+/−^: the protonated or deprotonated fragment ion of A-ring of polyphenols; i and j represent the position of the broken bond on C-ring. ^i,j^B^+/−^: the protonated or deprotonated fragment ion of B-ring of polyphenols. Glc: glucoside. Gal: galactoside. Chemical standard: These polyphenols were identified by comparing the *m/z* of precursor, retention time, and fragmentation pattern with standard compounds. The comparisons of mass spectra are available in Figure S1. Fragmentation pattern in reference [27]: These polyphenols were annotated through the manually verified fragmentation patterns in published literature. The mass spectra of all polyphenols are available in Figure S2.

## Data Availability

The data supporting the findings of this study are available in the Supplementary Materials.

## Supplementary Materials

The following supporting information can be downloaded at: https://www.mdpi.com/article/10.3390/antiox12040912/s1, Figure S1: The comparison of mass spectra between domestication-related polyphenols in soybean samples and chemical standards; Figure S2: The mass spectra of domestication-related polyphenols; Figure S3: The significant correlations between domestication-related polyphenols and total antioxidant capacities of soybeans; Figure S4: The mass spectra of malonylglycosylated flavonoids and isoflavonoids; Figure S5: The mass spectra of acetylglycosylated flavonoids and isoflavonoids; Table S1: The detailed information of domestication-related polyphenols; Table S2: The total antioxidant capacities of diverse soybean varieties determined by the FRAP and ABTS assays. Table S3: The detailed information on the malonylglycosylated and acetylglycosylated conjugates of polyphenols.
