# Supplementary material for: Metabolomic Analysis Reveals Domestication-Driven Reshaping of Polyphenolic Antioxidants in Soybean Seeds
Source: Antioxidants (Basel). 2023 Apr 11;12(4):912. doi: 10.3390/antiox12040912 (PMC10135580; doi:10.3390/antiox12040912)
Supplement: Supplementary file 1 [file antioxidants-12-00912-s001.zip › Table S2.pdf]

**Table S2 The total antioxidant capacities of diverse soybean varieties determined by the FRAP and ABTS assays.**

| Soybean variety | Species | Total antioxidant capacity (FRAP, µmol Trolox/g) | Total antioxidant capacity (ABTS, %) |
|---|---|---|---|
| Qi huang No.34 | *Glycine max* | 4.886 | 27.640 |
| Yu dou No.22 | *Glycine max* | 3.587 | 21.330 |
| Wan dou No.28 | *Glycine max* | 4.280 | 29.475 |
| Ji dou No.17 | *Glycine max* | 3.585 | 25.038 |
| Jin dou No23 | *Glycine max* | 4.494 | 35.670 |
| Tong shan tian e dan | *Glycine max* | 3.422 | 33.673 |
| W1 | *Glycine soja* | 11.992 | 74.751 |
| W11 | *Glycine soja* | 4.907 | 52.028 |
| W55 | *Glycine soja* | 6.886 | 52.464 |
| W61 | *Glycine soja* | 8.059 | 51.997 |
| W71 | *Glycine soja* | 8.621 | 54.054 |
| W92 | *Glycine soja* | 6.384 | 55.444 |
