# Supplementary material for: Metabolomic Analysis Reveals Domestication-Driven Reshaping of Polyphenolic Antioxidants in Soybean Seeds
Source: Antioxidants (Basel). 2023 Apr 11;12(4):912. doi: 10.3390/antiox12040912 (PMC10135580; doi:10.3390/antiox12040912)
Supplement: Supplementary file 1 [file antioxidants-12-00912-s001.zip › Table S3.pdf]

Table S3 The detailed information on the malonylglycosylated and acetylglycosylated conjugates of polyphenols.

Name	Exact mass	Formula	Characteristic fragment ions	Neutral loss	Ref
liquiritigenin-malonylglucoside	504.1268	$C_{24}H_{24}O_{12}$	[aglycone+H] $^{+}$ $C_{15}H_{13}O_4$ **257.0814**, [aglycone+H–B ring] $^{+}$ $C_9H_7O_3$ **163.0395**, $^{1,4}B^{+}$ $C_9H_7O_2$ **147.0446**, $^{1,3}A^{+}$ $C_7H_5O_3$ **137.0239**	248.0532	[45]
naringenin-malonylglucoside	520.1217	$C_{24}H_{24}O_{13}$	[aglycone+H] $^{+}$ $C_{15}H_{13}O_5$ **273.0763**, $^{1,3}A^{+}$ $C_7H_5O_4$ **153.0188**, $^{1,4}B^{+}$ $C_9H_7O_2$ **147.0446**	248.0532	[46]
pinocembrin-malonylglucoside	504.1268	$C_{24}H_{24}O_{12}$	[aglycone+H] $^{+}$ $C_{15}H_{13}O_4$ **257.0814**, $^{1,3}A^{+}$ $C_7H_5O_4$ **153.0188**, $^{1,4}B^{+}$ C_9H_7O **131.0497**	248.0532	[47]
hesperetin-malonylglucoside	550.1323	$C_{25}H_{26}O_{14}$	[aglycone+H] $^{+}$ $C_{16}H_{15}O_6$ **303.0869**, $^{1,4}B^{+}$ $C_{10}H_9O_3$ **177.0552**, $^{1,3}A^{+}$ $C_7H_5O_4$ **153.0188**, $^{1,4}B^{+}$–CH_2–H_2O $C_9H_5O_2$ **145.029**	248.0532	[48]
dihydrokaempferol-malonylglucoside	536.1166	$C_{24}H_{24}O_{14}$	[aglycone–H] $^{-}$ $C_{15}H_{11}O_6$ **287.0556**, [aglycone–H–H_2O] $^{-}$ $C_{15}H_9O_5$ **269.045**, [aglycone–H–CO] $^{-}$ $C_{14}H_{11}O_5$ **259.0606**, $^{0,2}A^{-}$ $C_8H_5O_4$ **165.0188**, $^{1,3}A^{-}$ $C_7H_3O_4$ **151.0031**, $^{1,4}A^{-}$ $C_6H_5O_3$ **125.0239**	248.0532	[49]
kaempferol-malonylglucoside	534.101	$C_{24}H_{22}O_{14}$	[aglycone+H] $^{+}$ $C_{15}H_{11}O_6$ **287.0556**, $^{0,2}A^{+}$ $C_8H_5O_4$ **165.0188**, $^{1,3}A^{+}$ $C_7H_5O_4$ **153.0188**, $^{0,3}A^{+}$ $C_7H_5O_3$ **137.0239**, $^{0,2}B^{+}$ $C_7H_5O_2$ **121.029**	248.0532	[50]
cyanidin-malonylglucoside	535.1088	$C_{24}H_{23}O_{14}{}^{+}$	[aglycone]$^{+}$ $C_{15}H_{11}O_6$ **287.0556**, $^{0,2}A^{+}$ $C_8H_5O_3$ **149.0239**, $^{0,2}B^{+}$ $C_7H_5O_3$ **137.0239**,	248.0532	[51]

Name	Exact mass	Formula	Characteristic fragment ions	Neutral loss	Ref
			$^{0,3}A^+$ *$C_7H_5O_2$* **121.029**		
catechin-malonylglucoside	538.1323	$C_{24}H_{26}O_{14}$	[aglycone+H] $^+$ *$C_{15}H_{15}O_6$* **291.0869**, $^{1,4}B^+$ *$C_9H_9O_3$* **165.0552**, $^{1,4}B^+$–H_2O *$C_9H_7O_2$* **147.0446**, $^{1,3}A^+$ *$C_7H_7O_3$* **139.0395**, $^{1,2}B^+$ *$C_7H_7O_2$* **123.0446**	248.0532	[27]
catechin-methyl ether-malonylglucoside	552.1479	$C_{25}H_{28}O_{14}$	[aglycone+H] $^+$ *$C_{16}H_{17}O_6$* **305.1025**, $^{1,2}A^+$–H_2O *$C_8H_7O_3$* **151.0395**, $^{1,4}B^+$–H_2O *$C_9H_7O_2$* **147.0446**, $^{1,3}A^+$ *$C_7H_7O_3$* **139.0395**, $^{1,2}B^+$ *$C_8H_9O_2$* **137.0603**	248.0532	[27]
daidzein-malonylglucoside	502.1111	$C_{24}H_{22}O_{12}$	[aglycone+H] $^+$ *$C_{15}H_{11}O_4$* **255.0657**, [aglycone+H–CO] $^+$ *$C_{14}H_{11}O_3$* **227.0708**, [aglycone+H–CO–CO] $^+$ *$C_{13}H_{11}O_2$* **199.0759**, $^{1,3}A^+$ *$C_7H_5O_3$* **137.0239**	248.0532	[52]
formononetin-malonylglucoside	516.1268	$C_{25}H_{24}O_{12}$	[aglycone+H] $^+$ *$C_{16}H_{13}O_4$* **269.0814**, [aglycone+H–CH_3] $^+$ *$C_{15}H_{10}O_4$* **254.0579**, [aglycone+H–CH_4] $^+$ *$C_{15}H_9O_4$* **253.0501**, [aglycone+H–CH_4O] $^+$ *$C_{15}H_9O_3$* **237.0552**, [aglycone+H–CO–CO] $^+$ *$C_{14}H_{13}O_2$* **213.0916**, $^{1,3}A^+$ *$C_7H_5O_3$* **137.0239**	248.0532	[52]
genistein-malonylglucoside	518.106	$C_{24}H_{22}O_{13}$	[aglycone+H] $^+$ *$C_{15}H_{11}O_5$* **271.0606**, [aglycone+H–CO] $^+$ *$C_{14}H_{11}O_4$* **243.065**, [aglycone+H–CO–CO] $^+$ *$C_{13}H_{11}O_3$* **215.0708**, $^{1,3}A^+$ *$C_7H_5O_4$* **153.0188**	248.0532	[52]
biochanin A-malonylglucoside	532.1217	$C_{25}H_{24}O_{13}$	[aglycone+H] $^+$ *$C_{16}H_{13}O_5$* **285.0763**, [aglycone+H–CH_3] $^+$ *$C_{15}H_{10}O_5$* **270.0528**,	248.0532	[52]

Name	Exact mass	Formula	Characteristic fragment ions	Neutral loss	Ref
			[aglycone+H–CH_4] $^+$ $C_{15}H_9O_5$ **269.045**, [aglycone+H–CH_4O] $^+$ $C_{15}H_9O_4$ **253.0501**, [aglycone+H–CO–CO] $^+$ $C_{14}H_{13}O_3$ **229.0865**, $^{1,3}A^+$ $C_7H_5O_4$ **153.0188**		
glycitein-malonylglucoside	532.1217	$C_{25}H_{24}O_{13}$	[aglycone+H] $^+$ $C_{16}H_{13}O_5$ **285.0763**, [aglycone+H–CH_3] $^+$ $C_{15}H_{10}O_5$ **270.0528**, [aglycone+H–CO] $^+$ $C_{15}H_{13}O_4$ **257.0814**, [aglycone+H–CH_3–CO] $^+$ $C_{14}H_{10}O_4$ **242.0579**, [aglycone+H–CO–CO] $^+$ $C_{14}H_{13}O_3$ **229.0865**, $^{1,3}A^+$ $C_8H_7O_4$ **167.0344**	248.0532	[52]
daidzein-dimalonylglucoside	588.1115	$C_{27}H_{24}O_{15}$	[aglycone+H] $^+$ $C_{15}H_{11}O_4$ **255.0657**, [aglycone+H–CO] $^+$ $C_{14}H_{11}O_3$ **227.0708**, [aglycone+H–CO–CO] $^+$ $C_{13}H_{11}O_2$ **199.0759**, $^{1,3}A^+$ $C_7H_5O_3$ **137.0239**	334.0536	[52]
genistein-dimalonylglucoside	604.1064	$C_{27}H_{24}O_{16}$	[aglycone+H] $^+$ $C_{15}H_{11}O_5$ **271.0606**, [aglycone+H–CO] $^+$ $C_{14}H_{11}O_4$ **243.065**, [aglycone+H–CO–CO] $^+$ $C_{13}H_{11}O_3$ **215.0708**, $^{1,3}A^+$ $C_7H_5O_4$ **153.0188**	334.0536	[52]
daidzein-glucoside-malonylglucoside	664.1639	$C_{30}H_{32}O_{17}$	[aglycone+H] $^+$ $C_{15}H_{11}O_4$ **255.0657**, [aglycone+H–CO] $^+$ $C_{14}H_{11}O_3$ **227.0708**, [aglycone+H–CO–CO] $^+$ $C_{13}H_{11}O_2$ **199.0759**, $^{1,3}A^+$ $C_7H_5O_3$ **137.0239**	248.0532+ 162.0528	[52]
genistein-glucoside-malonylglucoside	680.1589	$C_{30}H_{32}O_{18}$	[aglycone+H] $^+$ $C_{15}H_{11}O_5$ **271.0606**, [aglycone+H–CO] $^+$	248.0532+ 162.0528	[52]

Name	Exact mass	Formula	Characteristic fragment ions	Neutral loss	Ref
			$C_{14}H_{11}O_4$ **243.065**, [aglycone+H–CO–CO] $^+$ *$C_{13}H_{11}O_3$* **215.0708**, $^{1,3}A^+$ *$C_7H_5O_4$* **153.0188**		
genistein-malonylglucoside-pentoside	650.1483	$C_{29}H_{30}O_{17}$	[aglycone+H] $^+$ *$C_{15}H_{11}O_5$* **271.0606**, [aglycone+H–CO] $^+$ *$C_{14}H_{11}O_4$* **243.065**, [aglycone+H–CO–CO] $^+$ *$C_{13}H_{11}O_3$* **215.0708**, $^{1,3}A^+$ *$C_7H_5O_4$* **153.0188**	132.0423+ 248.0532	[52]
daidzein-malonylglucoside-malonylglucoside	750.1643	$C_{33}H_{34}O_{20}$	[aglycone+H] $^+$ *$C_{15}H_{11}O_4$* **255.0657**, [aglycone+H–CO] $^+$ *$C_{14}H_{11}O_3$* **227.0708**, [aglycone+H–CO–CO] $^+$ *$C_{13}H_{11}O_2$* **199.0759**, $^{1,3}A^+$ *$C_7H_5O_3$* **137.0239**	248.0532+ 248.0532	[52]
genistein-malonylglucoside-malonylglucoside	766.1593	$C_{33}H_{34}O_{21}$	[aglycone+H] $^+$ *$C_{15}H_{11}O_5$* **271.0606**, [aglycone+H–CO] $^+$ *$C_{14}H_{11}O_4$* **243.065**, [aglycone+H–CO–CO] $^+$ *$C_{13}H_{11}O_3$* **215.0708**, $^{1,3}A^+$ *$C_7H_5O_4$* **153.0188**	248.0532+ 248.0532	[52]
naringenin-acetylglucoside	476.1319	$C_{23}H_{24}O_{11}$	[aglycone+H] $^+$ *$C_{15}H_{13}O_5$* **273.0763**, $^{1,3}A^+$ *$C_7H_5O_4$* **153.0188**, $^{1,4}B^+$ *$C_9H_7O_2$* **147.0446**	204.0634	[46]
liquiritigenin-acetylglucoside	460.1369	$C_{23}H_{24}O_{10}$	[aglycone–H] $^-$ *$C_{15}H_{11}O_4$* **255.0657**, $^{1,3}A^-$ *$C_7H_3O_3$* **135.0082**, $^{1,3}B^-$ *C_8H_7O* **119.0497**	204.0634	[45]
hesperetin-acetylglucoside	506.1424	$C_{24}H_{26}O_{12}$	[aglycone–H] $^-$ *$C_{16}H_{13}O_6$* **301.0712**, $^{1,3}A^-$ *$C_7H_3O_4$* **151.0031**, $^{1,3}B^-$ *$C_9H_9O_2$* **149.0603**, $^{1,3}B^-$–CH_3 *$C_8H_6O_2$* **134.0368**	204.0634	[48]
kaempferol-acetylglucoside	490.1111	$C_{23}H_{22}O_{12}$	[aglycone+H] $^+$ *$C_{15}H_{11}O_6$* **287.0556**, $^{0,2}A^+$ *$C_8H_5O_4$* **165.0188**, $^{1,3}A^+$ *$C_7H_5O_4$* **153.0188**,	204.0634	[50]

Name	Exact mass	Formula	Characteristic fragment ions	Neutral loss	Ref
			$^{0,3}A^{+}$ $C_7H_5O_3$ **137.0239**, $^{0,2}B^{+}$ $C_7H_5O_2$ **121.029**		
catechin-acetylglucoside	494.1424	$C_{23}H_{26}O_{12}$	[aglycone–H] $^{-}$ $C_{15}H_{13}O_6$ **289.0712**, $^{1,2}A^{-}$–H_2O $C_8H_5O_3$ **149.0239**, $^{1,3}A^{-}$ $C_7H_5O_3$ **137.0239**, $^{1,2}B^{-}$ $C_7H_5O_2$ **121.029**	204.0634	[27]
daidzein-acetylglucoside	458.1213	$C_{23}H_{22}O_{10}$	[aglycone+H] $^{+}$ $C_{15}H_{11}O_4$ **255.0657**, [aglycone+H–CO] $^{+}$ $C_{14}H_{11}O_3$ **227.0708**, [aglycone+H–CO–CO] $^{+}$ $C_{13}H_{11}O_2$ **199.0759**, $^{1,3}A^{+}$ $C_7H_5O_3$ **137.0239**	204.0634	[52]
formononetin-acetylglucoside	472.1369	$C_{24}H_{24}O_{10}$	[aglycone+H] $^{+}$ $C_{16}H_{13}O_4$ **269.0814**, [aglycone+H–CH_3] $^{+}$ $C_{15}H_{10}O_4$ **254.0579**, [aglycone+H–CH_4] $^{+}$ $C_{15}H_9O_4$ **253.0501**, [aglycone+H–CH_4O] $^{+}$ $C_{15}H_9O_3$ **237.0552**, [aglycone+H–CO–CO] $^{+}$ $C_{14}H_{13}O_2$ **213.0916**, $^{1,3}A^{+}$ $C_7H_5O_3$ **137.0239**	204.0634	[52]
genistein-acetylglucoside	474.1162	$C_{23}H_{22}O_{11}$	[aglycone+H] $^{+}$ $C_{15}H_{11}O_5$ **271.0606**, [aglycone+H–CO] $^{+}$ $C_{14}H_{11}O_4$ **243.065**, [aglycone+H–CO–CO] $^{+}$ $C_{13}H_{11}O_3$ **215.0708**, $^{1,3}A^{+}$ $C_7H_5O_4$ **153.0188**	204.0634	[52]
glycitein-acetylglucoside	488.1319	$C_{24}H_{24}O_{11}$	[aglycone+H] $^{+}$ $C_{16}H_{13}O_5$ **285.0763**, [aglycone+H–CH_3] $^{+}$ $C_{15}H_{10}O_5$ **270.0528**, [aglycone+H–CO] $^{+}$ $C_{15}H_{13}O_4$ **257.0814**, [aglycone+H–CH_3–CO] $^{+}$ $C_{14}H_{10}O_4$ **242.0579**, [aglycone+H–CO–CO] $^{+}$ $C_{14}H_{13}O_3$ **229.0865**,	204.0634	[52]

Name	Exact mass	Formula	Characteristic fragment ions	Neutral loss	Ref
			$^{1,3}A^{+}$ *$C_8H_7O_4$* **167.0344**		
daidzein-diacetylrhamnoside	484.1369	$C_{25}H_{24}O_{10}$	[aglycone+H] $^{+}$ *$C_{15}H_{11}O_4$* **255.0657**, [aglycone+H–CO] $^{+}$ *$C_{14}H_{11}O_3$* **227.0708**, [aglycone+H–CO–CO] $^{+}$ *$C_{13}H_{11}O_2$* **199.0759**, $^{1,3}A^{+}$ *$C_7H_5O_3$* **137.0239**	230.079	[52]
genistein-diacetylrhamnoside	500.1319	$C_{25}H_{24}O_{11}$	[aglycone+H] $^{+}$ *$C_{15}H_{11}O_5$* **271.0606**, [aglycone+H–CO] $^{+}$ *$C_{14}H_{11}O_4$* **243.065**, [aglycone+H–CO–CO] $^{+}$ *$C_{13}H_{11}O_3$* **215.0708**, $^{1,3}A^{+}$ *$C_7H_5O_4$* **153.0188**	230.079	[52]
daidzein-acetyl-malonylglucoside	544.1217	$C_{26}H_{24}O_{13}$	[aglycone+H] $^{+}$ *$C_{15}H_{11}O_4$* **255.0657**, [aglycone+H–CO] $^{+}$ *$C_{14}H_{11}O_3$* **227.0708**, [aglycone+H–CO–CO] $^{+}$ *$C_{13}H_{11}O_2$* **199.0759**, $^{1,3}A^{+}$ *$C_7H_5O_3$* **137.0239**	290.0637	[52]
genistein-acetyl-malonylglucoside	560.1166	$C_{26}H_{24}O_{14}$	[aglycone+H] $^{+}$ *$C_{15}H_{11}O_5$* **271.0606**, [aglycone+H–CO] $^{+}$ *$C_{14}H_{11}O_4$* **243.065**, [aglycone+H–CO–CO] $^{+}$ *$C_{13}H_{11}O_3$* **215.0708**, $^{1,3}A^{+}$ *$C_7H_5O_4$* **153.0188**	290.0637	[52]

[aglycone+H] $^{+}$: the protonated ion of the aglycone of polyphenols.

[aglycone–H] $^{-}$: the deprotonated ion of the aglycone of polyphenols.

$^{i,j}A^{+/-}$: the protonated or deprotonated fragment ion of A-ring of polyphenols, i and j represent the position of the broken bond on C-ring.

$^{i,j}B^{+/-}$: the protonated or deprotonated fragment ion of B-ring of polyphenols.

These known and novel polyphenols were structurally characterized using the manually verified characteristic fragment ions of aglycone and featured neutral loss of modification groups based on published literature.

27. Li, H.-J.; Deinzer, M.L. Tandem Mass Spectrometry for Sequencing Proanthocyanidins. *Analytical Chemistry* **2007**, *79*, 1739-1748, doi:10.1021/ac061823v.

45. Fang, S.; Qu, Q.; Zheng, Y.; Zhong, H.; Shan, C.; Wang, F.; Li, C.; Peng, G. Structural characterization and identification of flavonoid aglycones in three Glycyrrhiza species by liquid chromatography with photodiode array detection and quadrupole time-of-flight mass spectrometry. *J Sep Sci* **2016**, *39*, 2068-2078, doi:10.1002/jssc.201600073.

46. Iijima, Y.; Suda, K.; Suzuki, T.; Aoki, K.; Shibata, D. Metabolite Profiling of Chalcones and Flavanones in Tomato Fruit. *Journal of the Japanese Society for Horticultural Science* **2008**, *77*, 94-102, doi:10.2503/jjshs1.77.94.

47. Pellati, F.; Orlandini, G.; Pinetti, D.; Benvenuti, S. HPLC-DAD and HPLC-ESI-MS/MS methods for metabolite profiling of propolis extracts. *J Pharm Biomed Anal* **2011**, *55*, 934-948, doi:10.1016/j.jpba.2011.03.024.

48. Zheng, G.D.; Zhou, P.; Yang, H.; Li, Y.S.; Li, P.; Liu, E.H. Rapid resolution liquid chromatography-electrospray ionisation tandem mass spectrometry method for identification of chemical constituents in Citri Reticulatae Pericarpium. *Food Chem* **2013**, *136*, 604-611, doi:10.1016/j.foodchem.2012.08.040.

49. Abad-Garcia, B.; Garmon-Lobato, S.; Berrueta, L.A.; Gallo, B.; Vicente, F. A fragmentation study of dihydroquercetin using triple quadrupole mass spectrometry and its application for identification of dihydroflavonols in Citrus juices. *Rapid Commun Mass Spectrom* **2009**, *23*, 2785-2792, doi:10.1002/rcm.4182.

50. Ma, C.; Lv, H.; Zhang, X.; Chen, Z.; Shi, J.; Lu, M.; Lin, Z. Identification of regioisomers of methylated kaempferol and quercetin by ultra high performance liquid chromatography quadrupole time-of-flight (UHPLC-QTOF) tandem mass spectrometry combined with diagnostic fragmentation pattern analysis. *Anal Chim Acta* **2013**, *795*, 15-24, doi:10.1016/j.aca.2013.07.038.

51. Barnes, J.S.; Schug, K.A. Structural characterization of cyanidin-3,5-diglucoside and pelargonidin-3,5-diglucoside anthocyanins: Multi-dimensional fragmentation pathways using high performance liquid chromatography-electrospray ionization-ion trap-time of flight mass spectrometry. *International Journal of Mass Spectrometry* **2011**, *308*, 71-80, doi:10.1016/j.ijms.2011.07.026.

52. Nakata, R.; Yoshinaga, N.; Teraishi, M.; Okumoto, Y.; Huffaker, A.; Schmelz, E.A.; Mori, N. A fragmentation study of isoflavones by IT-TOF-MS using biosynthesized isotopes. *Biosci Biotechnol Biochem* **2018**, *82*, 1309-1315, doi:10.1080/09168451.2018.1465810.
