# Supplementary material for: Metabolomic Analysis Reveals Domestication-Driven Reshaping of Polyphenolic Antioxidants in Soybean Seeds
Source: Antioxidants (Basel). 2023 Apr 11;12(4):912. doi: 10.3390/antiox12040912 (PMC10135580; doi:10.3390/antiox12040912)
Supplement: Supplementary file 1 [file antioxidants-12-00912-s001.zip › Supplementary_Figures.pdf]

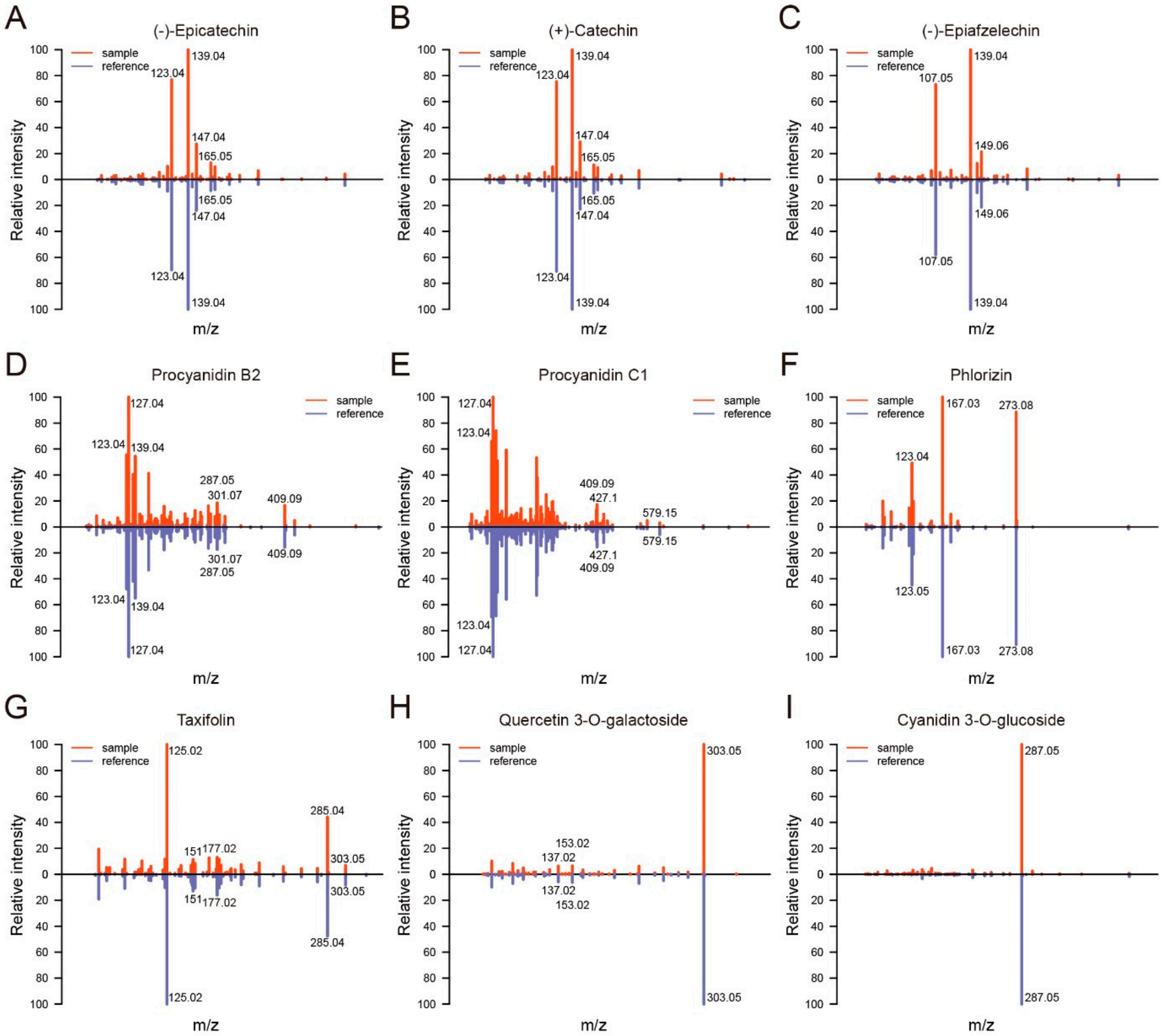

Figure S1 The comparison of mass spectra between domestication-related polyphenols in soybean samples and chemical standards.

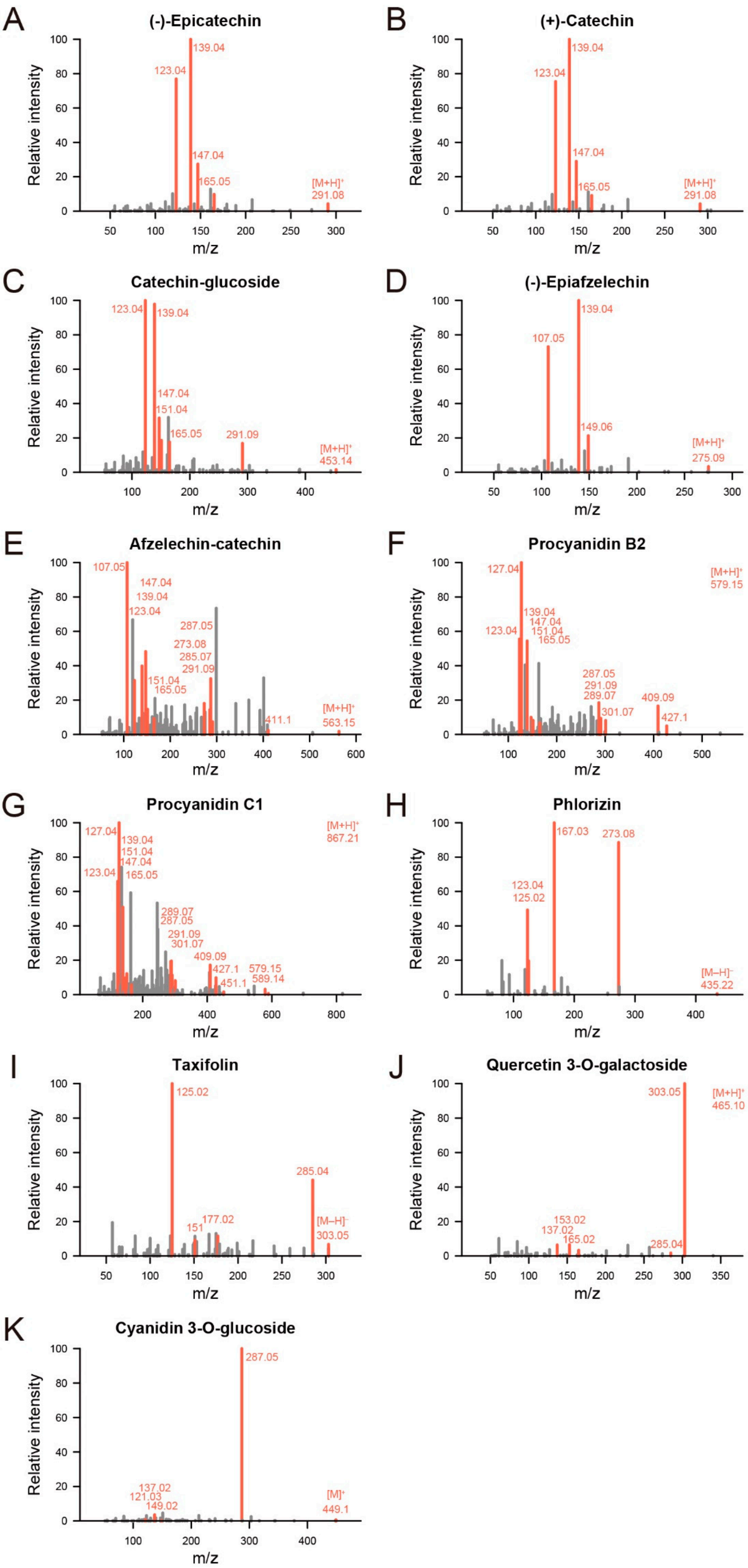
A
(-)-Epicatechin
Relative intensity
m/z
123.04
139.04
147.04
165.05
[M+H]+
291.08
B
(+)-Catechin
123.04
139.04
147.04
165.05
[M+H]+
291.08
C
Catechin-glucoside
123.04
139.04
147.04
151.04
165.05
291.09
[M+H]+
453.14
D
(-)-Epiafzelechin
107.05
139.04
149.06
[M+H]+
275.09
E
Afzelechin-catechin
107.05
147.04
139.04
123.04
287.05
273.08
285.07
291.09
151.04
165.05
411.1
[M+H]+
563.15
F
Procyanidin B2
127.04
[M+H]+
579.15
139.04
147.04
151.04
165.05
123.04
287.05
291.09
289.07
301.07
409.09
427.1
G
Procyanidin C1
127.04
139.04
151.04
147.04
123.04
165.05
[M+H]+
867.21
289.07
287.05
291.09
301.07
409.09
427.1
451.1
579.15
589.14
H
Phlorizin
167.03
273.08
123.04
125.02
[M–H]−
435.22
I
Taxifolin
125.02
285.04
151
177.02
[M–H]−
303.05
J
Quercetin 3-O-galactoside
303.05
[M+H]+
465.10
153.02
137.02
165.02
285.04
K
Cyanidin 3-O-glucoside
287.05
137.02
121.03
149.02
[M]+
449.1

Figure S2 The mass spectra of domestication-related polyphenols.

(A) The mass spectra of (-)-epicatechin. (B) The mass spectra of (+)-catechin. (C) The mass spectra of catechin-glucoside. (D) The mass spectra of (-)-epiafzelechin. (E) The mass spectra of afzelechin-catechin. (F) The mass spectra of procyanidin B2. (G) The mass spectra of procyanidin C1. (H) The mass spectra of phlorizin. (I) The mass spectra of taxifolin. (J) The mass spectra of quercetin 3-O-galactoside. (K) The mass spectra of cyanidin 3-O-glucoside. The characteristic fragment ions of each mass spectra were labeled in red.

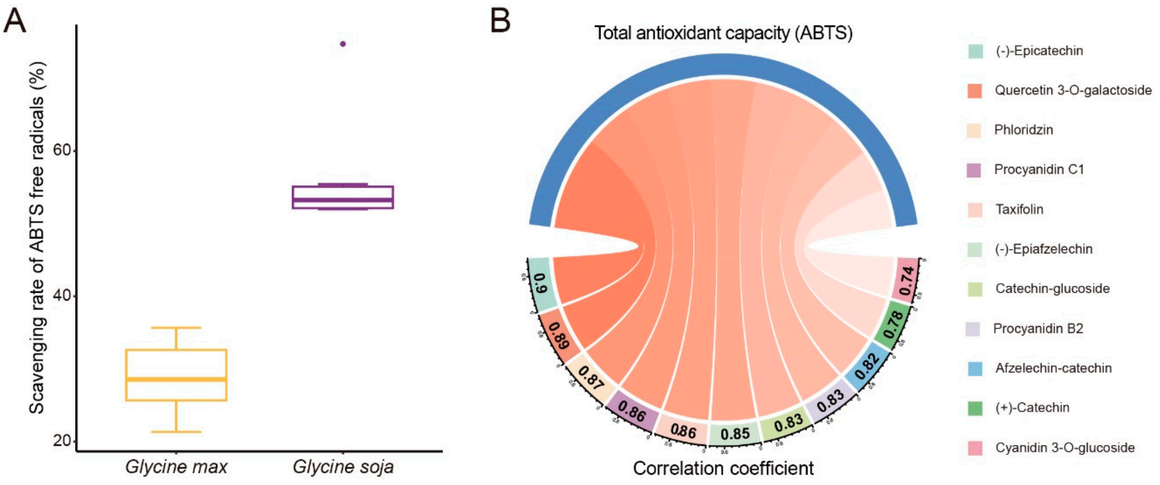

Figure S3 The significant correlations between domestication-related polyphenols and total antioxidant capacities of soybeans.

(A) The significant variation of total antioxidant capacity determined by the ABTS assay between wild (*Glycine soja*) and cultivated soybean varieties (*Glycine max*). Student's t-Test was used. (B) The chord diagram of strong positive correlative relationship between each domestication-related polyphenols and total antioxidant capacities determined by the ABTS assay.

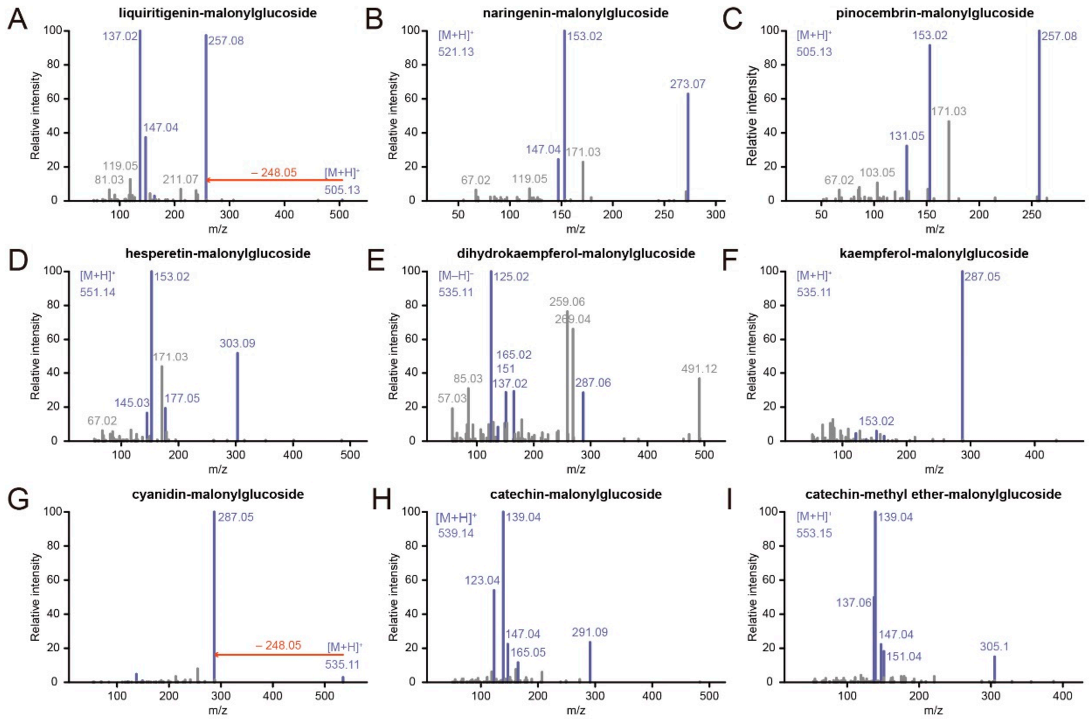

Figure S4 The mass spectra of malonylglycosylated flavonoids and isoflavonoids.

(A) The mass spectra of liquiritigenin-malonylglucoside. (B) The mass spectra of naringenin-malonylglucoside. (C) The mass spectra of pinocembrin-malonylglucoside. (D) The mass spectra of hesperetin-malonylglucoside. (E) The mass spectra of dihydrokaempferol-malonylglucoside. (F) The mass spectra of kaempferol-malonylglucoside. (G) The mass spectra of cyanidin-malonylglucoside. (H) The mass spectra of catechin-malonylglucoside. (I) The mass spectra of catechin-methyl ether-malonylglucoside. The characteristic fragment ions of each mass spectra were labeled in blue.

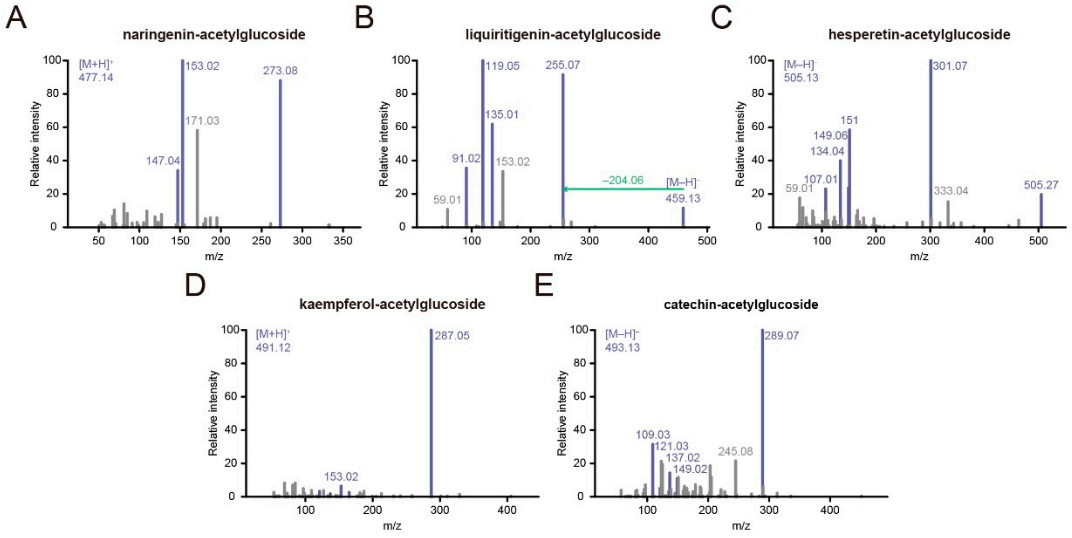

Figure S5 The mass spectra of acetylglycosylated flavonoids and isoflavonoids.

(A) The mass spectra of naringenin-acetylglucoside. (B) The mass spectra of liquiritigenin-acetylglucoside. (C) The mass spectra of hesperetin-acetylglucoside. (D) The mass spectra of kaempferol-acetylglucoside. (E) The mass spectra of catechin-acetylglucoside. The characteristic fragment ions of each mass spectra were labeled in blue.
